# Obtaining Microcellulose from Solid Agro-Waste by Ball Mill Assisted by Light Acid Hydrolysis Process

**DOI:** 10.1021/acsomega.4c07196

**Published:** 2025-01-01

**Authors:** Priscila S. Souza, Cristiani V. B. Grisi, Érica C. Monção, Marcus V. S. da Silva, Antonia L. de Souza

**Affiliations:** † Postgraduate Program in Chemistry, Universidade Federal da Paraíba, Cidade Universitária, João Pessoa 58051-900, Brazil; ‡ Postgraduate Program in Chemical engineering, Universidade Federal da Paraíba, Cidade Universitária, João Pessoa 58051-900, Brazil; § Postgraduate Program in Food Science and Technology, Universidade Federal da Paraíba, Cidade Universitária, João Pessoa 58051-900, Brazil; ∥ Postgraduate Program in Physics, Universidade Federal da Bahia, Campus Universitário de Ondina - Ondina, Salvador 40170-115, Brazil

## Abstract

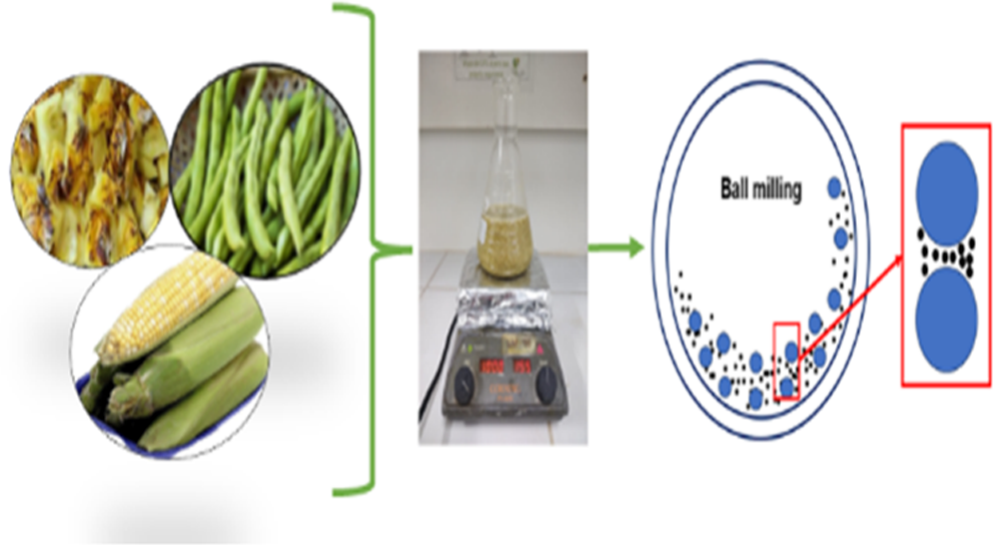

Cellulose, the most abundant biopolymer on Earth, is biodegradable, nontoxic, and derived from renewable sources. Its properties and applications depend on the extraction methods and sources, making plant waste reuse a sustainable production option. This study aimed to assess the potential of cowpea pod skin (*Vigna unguiculata*) as a source of microcellulose (CPMC) using a chemical-mechanical process involving ball milling combined with acid hydrolysis. For a comparative analysis of the method’s efficiency and biomass performance, corn straw (*Zea mays* L., CSMC) and pineapple peel (*Ananas comosus*, PPMC) were also utilized as extraction sources. The chemical composition of microcelluloses (MCs) was investigated by Fourier Transform Infrared Spectroscopy (FTIR), thermal behavior by Thermogravimetric Analysis (TGA), crystallinity by X-ray Diffraction (XRD), morphologies by Scanning Electron Microscopy (SEM), and shape and size by Atomic Force Microscopy (AFM). In the FTIR spectra, absorption bands characteristic of cellulose were observed at 3408 cm^–1^ (hydroxyl group OH stretching), 1640 cm^–1^ (adsorbed water molecules), 1205 cm^–1^ (O–H deformation vibration), 1165 cm^–1^ (C–O–C– stretching vibration), 1113 cm^–1^ (glucose ring stretching vibration), 1055 cm^–1^ (CO stretching), 1028 cm^–1^ (C–OH stretching), and 895 cm^–1^ (β-glycosidic bonds). The TGA/DTG curves of all the samples showed three stages of mass loss, and CPMC proved to be the sample with the greatest thermal stability. The crystallinity indices of the MCs samples ranged between 69.23–75%. The micrographs show compact and lamellar materials. However, AFM measurements revealed distinct nanostructures for each of the MCs obtained, displaying lamellar structures from 20 to 280 nm. Therefore, this method was efficient for extracting MCs from different types of biomass. The analyses demonstrated greater efficiency in the CPMC and CSMC samples. In this context, they have become promising candidates for application in a wide range of industrial materials.

## Introduction

Organic plant materials account for approximately 40–50% of the waste discarded into the environment and have been extensively utilized as a source of lignocellulosic material. This approach not only promotes reuse to mitigate the environmental impacts of improper disposal but also supports the development of more sustainable materials. Given that agricultural residues represent a significant source of cellulose biomass, considerable research has focused on extracting cellulose from various agricultural byproducts, including vegetable and fruit residues.^1,2^

Cellulose has garnered significant attention as the most abundant polymer on Earth, primarily found in the cell walls of plants. It exhibits exceptional properties such as biodegradability and biocompatibility, making it highly versatile. This biopolymer consists of long chains of β-d-glucopyranose units interconnected through β-1,4-glycosidic bonds, forming a repeating dimeric structure.^3^

In the industrial sector, cellulose is predominantly utilized in smaller fractions at the micro and nanoscales through the isolation of nanocellulose (NC) or microcellulose (MC). The study of NC/MC has acquired scientific attention, as it can be used in several areas, such as food, mechanical engineering, biomedicine, and health. NC and MC enable the production of various morphologies such as fibers, crystals, rods, and whiskers, each characterized by distinct properties and dimensions. These materials have applications in diverse fields, including food packaging, paper production, green composite manufacturing, and coating materials.^4^

The three main categories are nano/microfibrillated cellulose (NFC/MFC), nano/microcrystalline cellulose (NCC/MCC), and bacterial nano/microcellulose (NB/MB).^5^ There are several methodologies for the isolation of NC from cellulose fibers, including acid hydrolysis,^6^ homogenization,^7^ grinding,^8^ and mixed or chemical-mechanical method.^9^ To address the drawbacks associated with the chemical-mechanical processes for cellulose isolation, the literature suggests adopting combined processes. These methods are recognized as environmentally efficient approaches for the produce NFC/MFC.^10,11^

In the field of potential reuse of plant residues, pineapple (*Ananas camosus*), cowpea (*Vigna unguiculata*), and corn (*Zea mays* L.) species stand out, as they are among the most produced vegetables and are part of the Brazilian diet. Brazil is currently the second largest pineapple producer.^12,13^ Pineapple peels are composed of lignocellulosic structuring molecules with an estimated proportion of 24% cellulose, 29% hemicellulose, and 6% lignin.^14^ Brazil is the third largest producer in the world^15^ regarding to beans and corn. In the specific case of cowpea, the pods are rich in cellulose (29.97%), hemicellulose (24.33%), and lignin (7.31%).^16^ However, corn straw can reach 35–40% cellulose, 21–25% hemicellulose and 11–19% lignin.^17^ The consumption of these vegetables generates exorbitant amounts of waste from peels, straws, and skins, which are neglected.

The literature on NC/MC extraction is quite vast, and recently, the combination of chemical-mechanical processes to obtain NC/MC has been studied, such as NFC isolated from stems of sunflower;^18^ NFC isolated from leaf fiber of *Ananas comosusN36*,^19^ extraction of NFC from pomelo peel,^20^ generation of NFC from banana peel,^21^ use of corn cobs (*Z. mays* spp.) to isolate NC;^22^ NC produced from raw cellulose or tomato bagasse;^23^ lignin-cellulose nanocrystals from hemp;^24^ structural, morphological, and thermal properties of NFC from Napier fiber (*Pennisetum purpureum*);^25^ and nanocellulose isolated from eucalyptus.^26^

The proposed hypothesis suggests that optimizing the ball milling process is an effective method for extracting cellulose from various plant biomass residues, including pineapple peel, cowpea pod skin, and corn straw. To our knowledge, no studies have identified the extraction of NC/MC from cowpea pod skin. Therefore, the objective of this study was to extract MC from corn straw, pineapple peels, and cowpea pod skin using a ball mill assisted by an acid hydrolysis process and to analyze the morphology, thermal stability, crystallinity, and chemical composition of the resulting material.

## Material and Methods

### Material

Cowpea pods (*V. unguiculata*), corn (*Z. mays* L.) and pineapple (*A. camosus*) were purchased in the period January–April, 2022, in open-air markets in the municipalities of Campina Grande and João Pessoa, Paraíba, Brazil. The vegetables were cleaned with detergent and running water and then selected according to the physical integrity of the peels, skins and straws. Pineapple peel was removed using a knife. The cowpea pod skins were manually removed by separating the skin and grains. Corn straw was obtained by manually separating the cob straw from corn. The samples were then spread on a metal tray forming a thin layer and dried in an oven with air circulation at 50 °C until a constant mass was obtained.^27^ After drying, the samples were crushed in a domestic blender to reduce the particle size. At the end of the process, they were vacuum packed, placed away from light and stored in a refrigerator at 4 °C, where they remained until subsequent stages.

PA grade reagents were purchased and used without any purification: Anhydrous Ethyl Alcohol (99.8%) from Dinâmica; Hydrogen Peroxide (35%) from Synth; Sulfuric Acid (98%) from α, and sodium hydroxide P.A. from Synth.

### Extraction of Microcellulose

The process of obtaining microcellulose from peels, skins and straws was carried out according to the methodology described by Plermjai et al.,^28^ with modifications in the grinding stage (diameter of the balls, extraction time, concentration, and volume of sulfuric acid). The process was divided into three stages, the first consisted of the delignification (partial elimination of lignin and hemicellulose) of the biomass, the second was the extraction of cellulose, and the third was the obtaining of microcellulose.

### Delignification

A total of 20 g of each sample was added to 200 mL (1:1 v/v) of ethanol/distilled water and left under magnetic stirring for 2 h at the boiling temperature. After this period, the mixture was filtered under vacuum, and the process was repeated to ensure that the waxes were removed. After removing the wax, 200 mL of 5% aqueous sulfuric acid solution was added to the resulting powder. The reaction mixture was stirred for 2 h at 60 °C, followed by filtration, washing of the solid material with an ethanol/water solution (1:1), and vacuum filtration. The process was repeated four times to partially remove lignin and hemicellulose, after which it was washed with distilled water until a neutral pH was achieved. The resulting solid material was dried in an oven under air circulation at 60 °C until a constant mass was obtained.

### Cellulose Extraction

The materials obtained after delignification were bleached using 24% hydrogen peroxide (v/v) and 4% sodium hydroxide (m/v) in an aqueous solution at a proportion of (1:10, g/mL). The reaction was carried out under mechanical agitation at room temperature (25 °C) for 2 h to ensure the removal of lignin and hemicellulose. At the end of the extraction, the residue was filtered using a vacuum pump and washed with distilled water until neutral pH was reached. The obtained cellulose was dried in an oven with air circulation at 60 °C until a constant mass was obtained.

### Obtaining Microcellulose

Microcellulose was prepared using ball milling and acid hydrolysis. Cellulose (3 g) was placed in a polyethylene container (200 mL), 90 mL of 30% aqueous sulfuric acid solution, and 150 g of 3 mm diameter zirconia balls (50:1, m/m). Milling was performed for 15 h. After this period, the balls were removed and the milled product was filtered to remove the acid and washed with distilled water until a neutral pH was obtained. The samples were then dried in an oven with air circulation at 60 °C until a constant mass was obtained. The obtained microcellulose was stored under refrigeration (4 °C) until characterization.

### Yield Calculation

The overall yield of the MFCs extraction processes is expressed in percentage terms as the ratio between the mass of oven-dried MFCs (*M*_f_) and the mass of fresh biomass (*M*_i_), according to eq 1. The yield of the extraction of microcelluloses from celluloses obtained after chemical treatment is given by the ratio between the mass of oven-dried CMFs (*M*_f_) and the mass of celluloses (*M*_c_), eq 2.
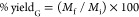
1
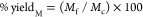
2

### Characterization of the Samples

Characterization analyses were carried out for the vegetable residues corn straw, cowpea pod skin, and pineapple peel *in natura* and after the other treatment stages (Table 1).

**Table 1:** Samples Code and Respective Treatment

samples	treatment
PPWT	pineapple peel without treatment
PPT1	pineapple peel after acid treatment
PPT2	pineapple peel after basic treatment
PPMC	microcellulose pineapple peel
CPWT	cowpea pod skin without treatment
CPT1	cowpea pod skin after acid treatment
CPT2	cowpea pod skin after basic treatment
CPMC	microcellulose cowpea pod skin
CSWT	corn straw without treatment
CST1	corn straw after acid treatment
CST2	corn straw after basic treatment
CSMC	microcellulose corn straw

The samples were characterized by the methods of: Fourier Transform Infrared Spectroscopy (FTIR), Thermogravimetric Analysis and its derivative (TGA/DTG), X-ray Diffraction (XRD), Scanning Electron Microscopy (SEM) and Atomic Force Microscopy (AFM).

### Fourier Transform Infrared Spectroscopy (FTIR) Analysis

The absorption spectra in the infrared region were obtained on a Shimadzu IR Prestige-21 spectrophotometer between 4000 and 400 cm^–1^ using a potassium bromide tablet. For the KBr analyses, 1 mg of the sample was mixed and homogenized with 99 mg of KBr in an agate mortar. This mixture was compacted in a hydraulic press at 80 kgf to form a tablet, which was used for the insertion and reading of the equipment.

### Thermogravimetric Analysis (TGA/DTG)

Thermogravimetric curves (TGA/DTG) of the synthesized samples were obtained using a Shimadzu thermogravimetric analyzer (model TGA 60/60H), using an alumina sample holder, flow of 50 mL/min of N_2_ with a heating rate of 10 °C/min, and a range of 30–600 °C.

### Scanning Electron Microscopy (SEM)

The morphological aspects of the samples were analyzed using Scanning Electron Microscopy (SEM; TESCAN brand, model MIRA3). The powders were deposited on double-sided carbon tape glued to an aluminum sample holder, covered with a gold film to act as a conductive medium, and then analyzed with an acceleration voltage of 5 kV.

### Atomic Force Microscopy (AFM)

Atomic force microscopy (AFM) was performed using a Shimadzu SPM-9700 in phase mode (noncontact) with a super sharp probe with a curvature radius of less than 5 nm. The samples were deposited on freshly cleaved muscovite mica slides from a suspension prepared in ultrapure water.

### X-ray Diffraction (XRD)

X-ray diffraction was carried out using an XRD-6000 diffractometer (Shimadzu, Tokyo, Japan) with a power of 2 kVA, voltage of 30 kV, current of 30 mA, and Kα radiation from copper (0.154 nm). The scans were performed in the range of 10–60° with a step of 0.01° and a speed of 1.7° s^–1^. The diffractograms were separated into a halo representing the contribution from the amorphous region, and peaks representing the crystalline regions of the polymer.

The crystallinity index (CI) was calculated using eq 3, subtracting the maximum diffraction intensity (22° < 2Θ < 23°) that represents the crystalline material (*I*_c_), from the minimum diffraction intensity (18° < 2Θ < 19°) that represents the amorphous material (*I*_am_). In the diffractogram obtained from cellulosic compounds, the crystalline part of the material is represented by the height of the highest peak and the amorphous part refers to the minimum height between the peaks. The CI is the difference between these two intensities divided by the intensity of the most intense peak, as shown in eq 3 Segal et al.^29^
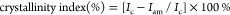
3

## Results and Discussion

### Yield

Table 2 shows the global yield (Yield_G_) and yield to the step to obtain microcelluloses (Yield_M_) from the celluloses extracted from biomass.

**Table 2:** Extraction Process Yield Results^a^

biomass	yield_G_ (%)	yield_M_ (%)
cowpea pod skin	30.54	81.0
pineapple peel	8.17	57.3
corn straw	22.54	75.9

a Yield_G_: Overall yield of the MFCs extraction processes expressed in percentage; Yield_M_: yield of the extraction of microcelluloses from celluloses obtained.

The overall yield corresponds to the percentage of mass obtained in the preparation of microcellulose after all stages of the process. The values found are in accordance with what is reported in the literature for the composition of each biomass, since pineapple peels have around 24% cellulose in their composition, cowpea pods have 29.97% cellulose and corn straw can reach 35–40%.^14,16,17^ The yields obtained using only microcellulose were considered satisfactory when compared with literature data, mainly with respect to pineapple peel and corn straw biomass. For Cowpea pods skin biomass there are no reports in the literature to establish comparison.^26,30,31^

### Characterization of Samples

#### Fourier Transform Infrared (FTIR) Spectroscopy

**Figure 1:**
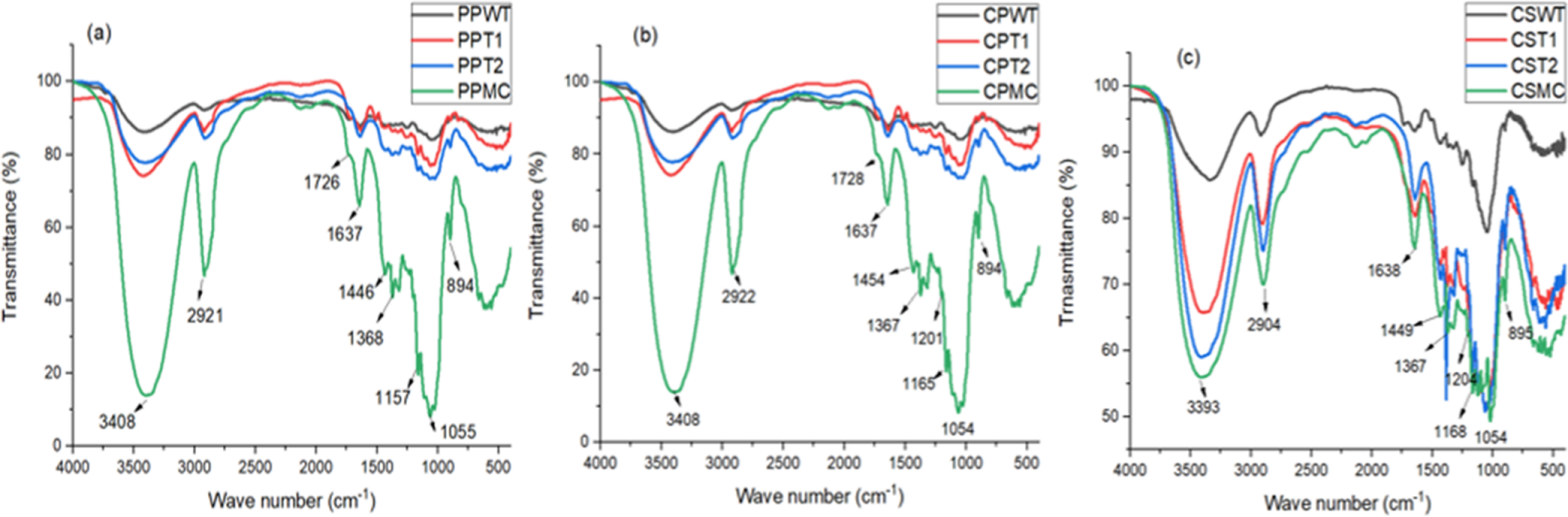
FTIR spectrum (a) pineapple peel, (b) cowpea pod skin and (c) corn straw. Pineapple peel without treatment (PPWT); pineapple peel after acid treatment (PPT1); pineapple peel after basic treatment (PPT2); microcellulose pineapple peel (PPMC); cowpea pod skin without treatment (CPWT); cowpea pod skin after acid treatment (CPT1); cowpea pod skin after basic treatment (CPT2); microcellulose cowpea pod skin (CPMC); corn straw without treatment (CSWT); corn straw after acid treatment (CST1); corn straw after basic treatment (CST2) and microcellulose corn straw (CSMC).

The FTIR spectra of pineapple peel, cowpea pod skin, and corn straw *in natura* and after each stage of the process used to obtain MCs are shown in Figure 1.

**Figure 2:**
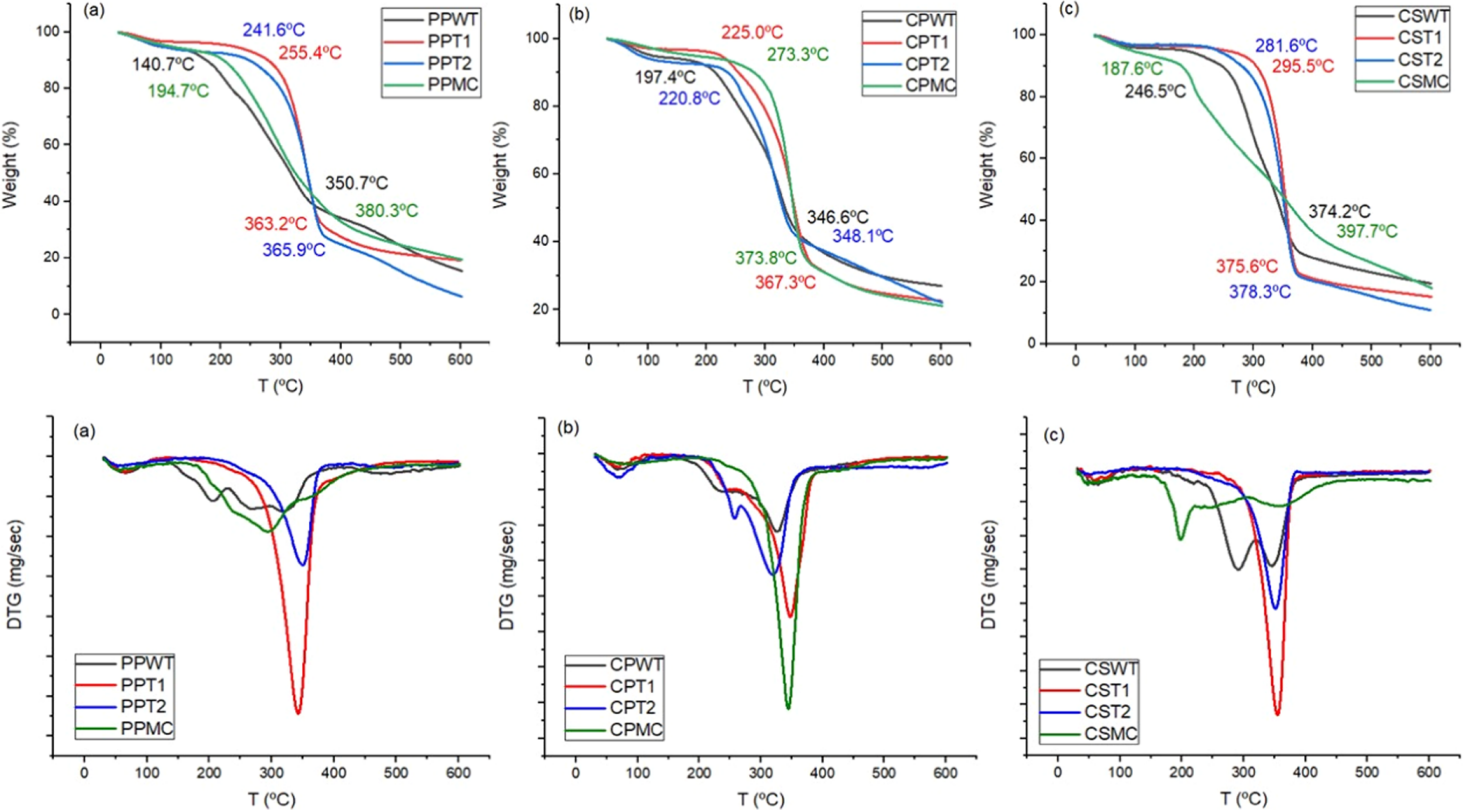
TGA and DTG curve of (a) pineapple peel, (b) cowpea pod skin and (c) corn straw. Pineapple peel without treatment (PPWT); pineapple peel after acid treatment (PPT1); pineapple peel after basic treatment (PPT2); microcellulose pineapple peel (PPMC); cowpea pod skin without treatment (CPWT); cowpea pod skin after acid treatment (CPT1); cowpea pod skin after basic treatment (CPT2); microcellulose cowpea pod skin (CPMC); corn straw without treatment (CSWT); corn straw after acid treatment (CST1); corn straw after basic treatment (CST2) and microcellulose corn straw (CSMC).

The characteristic peaks of cellulose, such as 3408 cm^–1^ (hydroxyl group OH stretching), 2920 cm^–1^ (CH– stretching vibration), 1640 cm^–1^ (adsorbed water molecules), 1445 cm^–1^ (CH– deformation vibration), 1367 cm^–1^ (CH– deformation vibration), 1319 cm^–1^ (H–C–H), 1205 cm^–1^ (O–H deformation vibration), 1165 cm^–1^ (C–O–C – stretching vibration), 1113 cm^–1^ (glucose ring stretching vibration), 1055 cm^–1^ (CO stretching), 1028 cm^–1^ (C–OH stretching), and 895 cm^–1^ (β-glycosidic bonds), were observed in all samples, indicating that the chemical pretreatment and ball milling process did not alter the cellulose structure.^28,32,33^ The peaks at 1728 cm^–1^ (C=O, vibration of the carboxylic group) observed in Figure 2a–c are attributed to hemicellulose. The peaks 1600 cm^–1^ (C=O stretching vibration), 1243 cm^–1^ (C=O out of plane stretching vibration) and 830 cm^–1^ (bending of the C–H bond of the arenes) are attributed to the presence of lignin.^28,34−36^

Characteristic bands of lignin and hemicellulose were observed in fresh pineapple peel, cowpea pod skin, and corn straw. However, the band at 1726 cm^–1^ in CSMC, which corresponds to the vibration of the carboxylic group C=O (hemicellulose), disappeared; the band at 1600 cm^–1^ was not observed for PPMC and CSMC, corresponding to the stretching vibrations of C=O (lignin). In all MCs, the band corresponding to C=O outside the plane of stretching vibration (lignin) at 1243 cm^–1^ disappeared, as did the band of flexion of the C–H bond of arenes (lignin) at 830 cm^–1^. The disappearance of bands was attributed to these polysaccharides, evidence of the effectiveness of the treatment, and the similarity presented by the three infrared spectra. This can be considered a strong indication of the reproducibility of the method used to obtain MCs.

#### Thermogravimetric Analysis

The results obtained from the TGA and DTG analyses of pineapple peel, cowpea pod skin, and corn straw are shown in Figure 2.

According to the observations in the DTG curves, all analyzed samples present a first stage of mass loss at approximately 70 °C, which corresponds to the loss of moisture and light volatile compounds. Mass loss stages between 200 and 280 °C were observed for the samples (PPWT, PPMC, CPWT, CPT1, CPT2, CSWT, and CSMC), which can be attributed to the thermal depolymerization of hemicellulose. Another stage between 320 and 370 °C was observed for all samples; this peak corresponds to the decomposition of cellulose and lignin. The peak, at around 470 °C, was observed only for PPWT, and it can be attributed to the degradation of residual lignin present in this sample.^37−39^

These results are analogous to the extraction process of the cellulose utilized, mainly considering that pineapple peel was efficient, as it promotes changes in the degradation process of the samples, and it is possible to notice an increase in thermal stability in cellulose (PPT2).^39^ Furthermore, the disappearance of the degradation peaks characteristic of hemicellulose and lignin can be observed, corroborating the possible elimination of these components.

When observing the TGA curves, the Tonset of CPMC was higher than that of the other samples (CPWT, CPT1, and CPT2). This result corroborates the FTIR analysis of this sample, where the CPMC after the extraction process showed a greater number of bands characteristic of lignin and hemicellulose and the remaining presence of amorphous regions. This effect can be attributed to the higher thermal resistance of the lignin. When lignin is incorporated into nanocellulose, it imparts favorable properties such as enhanced thermal stability.^40^

The Tonset values for PPMC and CSMC were lower than those observed for samples from the earlier stages of the microcellulose extraction process. The DTG curves indicate that PPMC and CSMC exhibited degradation at lower temperatures than the samples from earlier processes. This may be attributed to the presence of sulfonated groups remaining on the surface of the material.^41,42^ In the hydrolysis reaction using sulfuric acid, the greater the interaction of the acid with cellulose, the better is the efficiency in degrading the amorphous part of the cellulose. However, the presence of sulfate groups derived from sulfuric acid can decrease the thermal resistance of the material. The greater presence of amorphous material, such as residual lignin, could coat cellulose, making it less accessible to acid attack.^43^

A study of pineapple leaf fibers subjected to chemical-mechanical treatment reported a maximum degradation peak at 344 °C.^44^ In another study, Tmax values ranging from 315 to 350 °C were reported for cellulose in both paper and powder forms.^45^ In another study, nanocellulose was effectively applied to protein isolate films to enhance the gel-forming properties of soy protein isolates. NC demonstrated positive results for the materials tested, highlighting its potential applications.^46,47^ These findings support the feasibility of utilizing the MFCs obtained in this study considering their favorable thermal characteristics.

#### Scanning Electron Microscopy (SEM)

**Figure 3:**
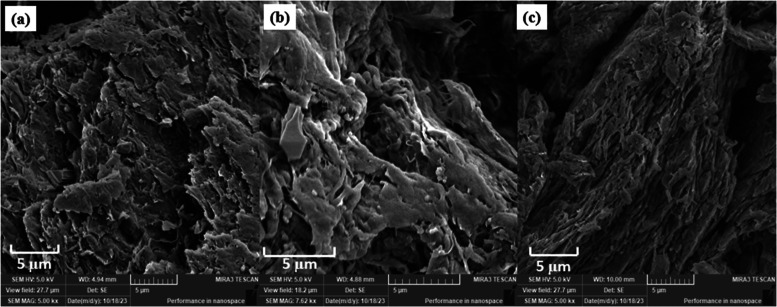
Micrographs obtained by SEM for (a) pineapple peel—PPMC, (b) cowpea pods skin—CPMC and (c) corn straw – CSMC.

The MC obtained after the process (PPMC, CPMC and CSMC) were analyzed using SEM to evaluate aspects of the resulting morphology, as well as to determine whether they fit into the MC category described in the literature. With the analysis, it is possible to observe the formation of agglomerates, suggesting a milling efficiency lower than desired. PPMC (Figure 3a) and CSMC (Figure 3c) showed a more lamellar characteristic morphology. The micrographs of all MCs showed porous morphology.

The MFCs of the materials studied (PPMC, CPMC, CSMC) are within the micrometric scale. The MCs obtained using the Eucalyptus, soybean straw, paper egg trays and paper industry waste by chemical-mechanical extraction showed compatible values with the ones reported in this study, tanging between ∼100–500 nm.^48−51^

Ball milling was used to extract MFC derived from cotton fabric scraps as a cross-linker to reinforce sodium alginate/chitosan hydrogels,^52^ and the technique was also used to extract cellulose from bamboo pulp, which was applied as reinforcement in geopolymer composites.^53^ The materials obtained by the authors had lengths above 100 nm, the same was observed for the MFCs obtained in this research, which shows a few of the possibilities of applying the materials produced with the process we used to produce MFCs from different types of biomasses.

#### Atomic Force Microscopy (AFM)

Although the obtained MCs presented lamellar structures, the fragmentation of these structures by dilution in water allowed for the evaluation of their basic nanostructures through AFM measurements, revealing significant variation among the different MCs analyzed. The lamellar structures previously observed by SEM are, in fact, composed of clusters of nanostructures with dimensions ranging from 20 to 280 nm.

**Figure 4:**
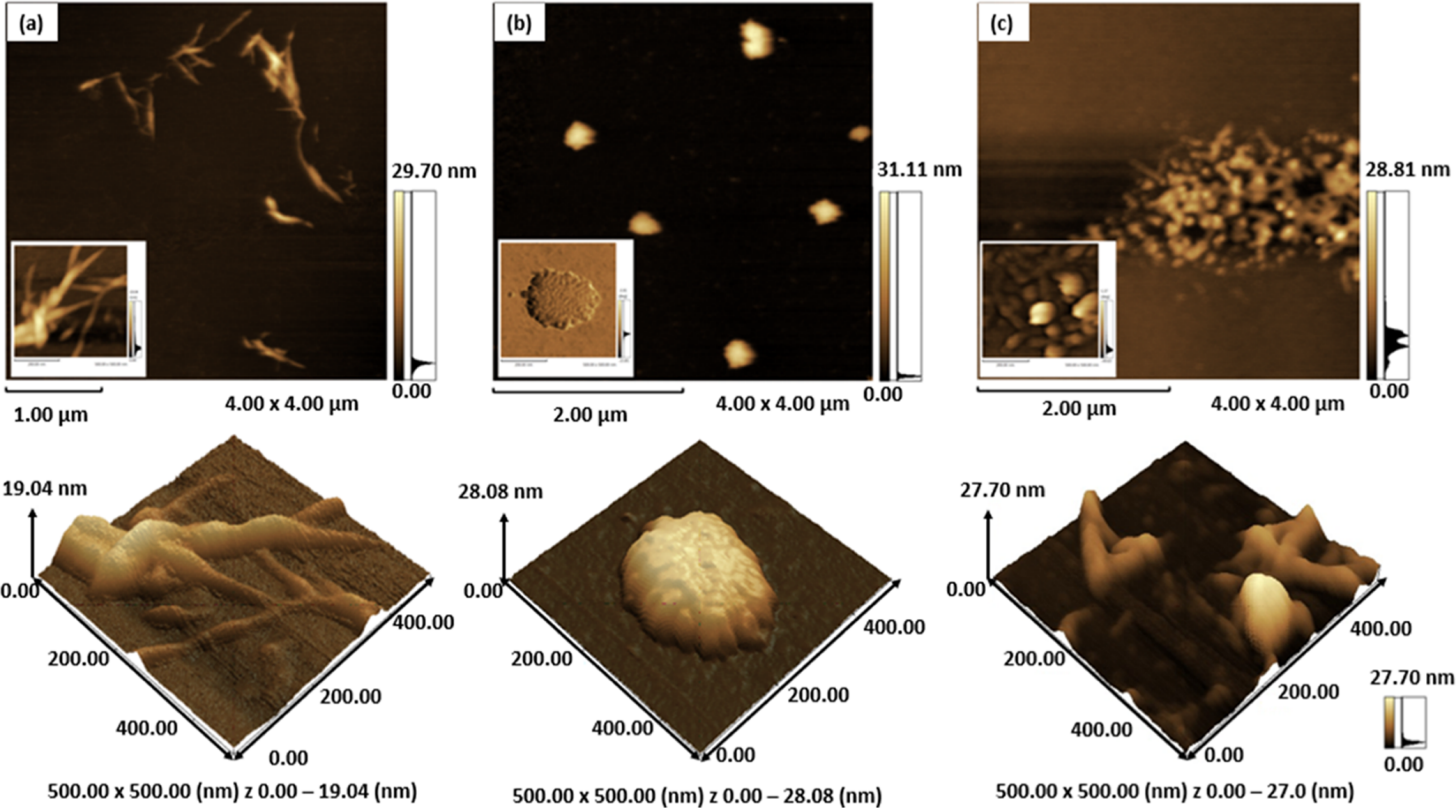
AFM image: height, 3D height and phase inserted (a) pineapple peel—PPMC, (b) cowpea pod skin—CPMC and (c) corn straw—CSMC.

The PPMC in Figure 4a, presents a structure of clusters of cellulose fibrils with lengths varying between 300 and 600 nm and widths ranging from 30 to 50 nm. In phase image (inserted), some globular domains are also observed, forming denser and more compact structures with spherical and elongated shapes. On the other hand, CPMC in Figure 4b presents a rounded structure with an average diameter of 280 nm, which is quite different from the structure of PPMC. However, the phase signal (inserted) reveals that these structures are composed of oblong nanoparticle clusters, with an average dimension of 30 nm along the major axis. Finally, CSMC in Figure 4c presents a structure with two interconnected but clearly distinct phases: spherical nanoparticles, some elongated, and nanocylinders, forming a tangle of nanocelluloses. The average dimensions of the nanoparticles are 100 nm, while the nanocylinders have lengths on the order of 250 nm and a cross-sectional width of 60 nm. All these structures presented by MCs bring with them unique properties and find a wide range of applications, confirming their great potential.^54,55^

#### X-ray Diffraction

**Figure 5:**
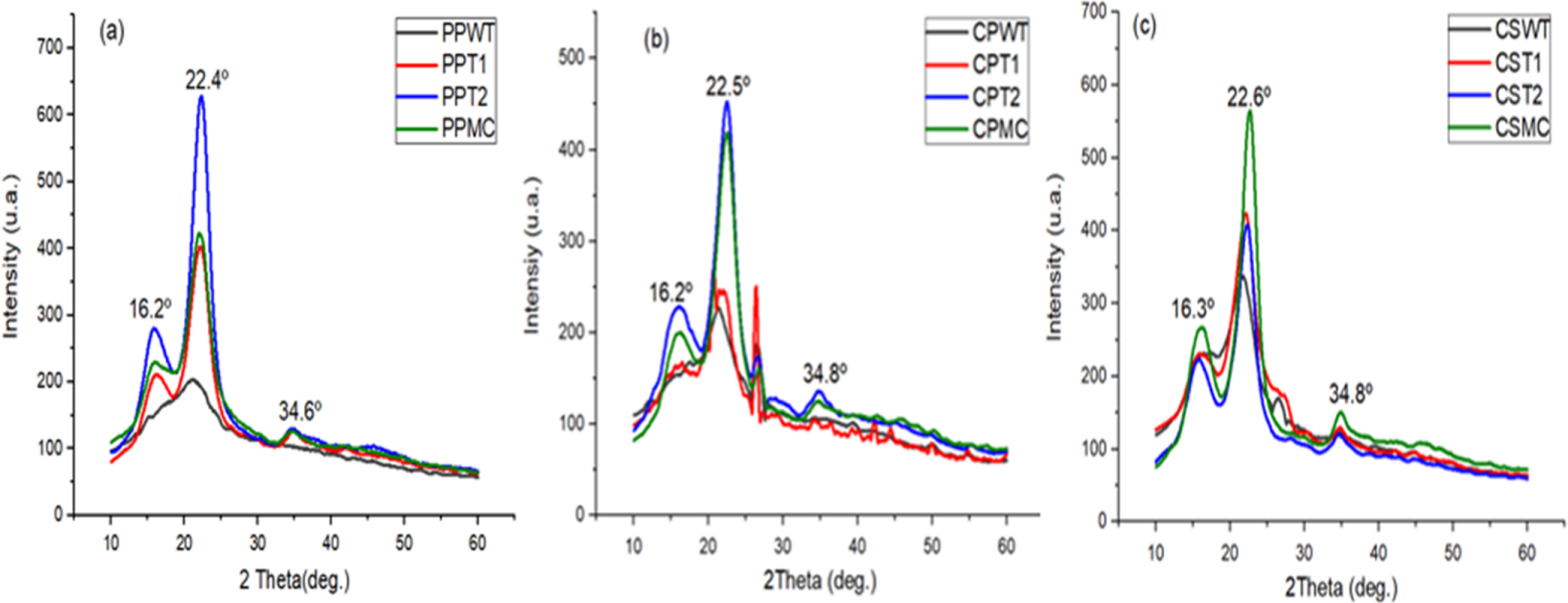
XRD spectrum (a) pineapple peel, (b) cowpea pod skin and (c) corn straw. Pineapple peel without treatment (PPWT); pineapple peel after acid treatment (PPT1); pineapple peel after basic treatment (PPT2); microcellulose pineapple peel (PPMC); cowpea pod skin without treatment (CPWT); cowpea pod skin after acid treatment (CPT1); cowpea pod skin after basic treatment (CPT2); microcellulose cowpea pod skin; (CPMC); corn straw without treatment (CSWT); corn straw after acid treatment (CST1); corn straw after basic treatment (CST2) and microcellulose corn straw (CSMC).

Figure 5 shows the XRD patterns of pineapple peel, cowpea pod skin, and corn straw in the fresh stages, after each treatment, and their respective MCs.

XRD analysis is a technique characterized by changes in the crystalline structure of the samples, and the results provide insights into the effect of the extraction process steps used to remove amorphous regions (lignin and hemicellulose) on the morphology of microcellulose aggregation.^56^ From the analysis of Figure 4, it can be seen that all *in natura* biomasses present only one peak at approximately 22.5° of greater prominence for CPWT and CSWT, and of lesser magnitude for PPWT. Regarding the samples after treatment (PPT1, PPT2, CPT1, CPT2, CST1, and CST2), diffraction peaks were observed at 2θ around 16.5, 22.5, and 34.5°, corresponding to the lattice planes (110), (200), and (004), which represent the typical structure of cellulose I.^57^ Similar results were observed in the extraction of NC from corn stem fibers,^37^ as well as in a study with acacia bark fibers.^58^

In this study, bleaching and acid hydrolysis did not affect the crystalline structure of cellulose, as no peaks with significant intensity characteristics of cellulose II and other cellulose polymorphs, were observed.^59^ From the XRD spectra, it is observed that the peaks at 2θ around 16.5° are absent or present less prominence in the *in natura* biomass, which reinforces the increase in the crystallinity of the material after the treatments are performed, and consequently the efficiency of the extraction method used. Additionally, the greater definition and intensity of the peak around 22° for the extracted cellulose, when compared to samples *in natura*, indicates an increase in crystallinity, suggesting that amorphous compounds such as hemicellulose and lignin were removed from the biomass.^39^

Cellulose I consist of a mixture of crystalline celluloses Iα and Iβ, with the Iβ form being predominant in plants. Celullose type II is a result of the modification of cellulose I. Cellulose types are characterized by characteristic peaks presented in analyses such as XRD. The X-ray diffraction pattern of cellulose-Iα (triclinic) shows three main peaks located at angles around 2θ = 16.50, 22.50, and 32.50°. The X-ray diffraction pattern of cellulose-Iβ (monoclinic) shows a shift in the main peaks located at angles 2θ of 15.20, 22.90, and 30.60°. Cellulose II showed diffraction peaks around 2θ = 12.27, 19.95, 22.13, and 34.53°. Unlike type II cellulose, standard cellulose is classified as cellulose type I, as it does not have a doublet in the main peak at 2θ = 23°.^60,61^

The biomass (PPWT and CSWT) presented the characteristic peaks observed for cellulose in the first chemical treatment (PPT1 and CST1), Table 3. However, there is a difference in the later steps in relation to the peak around 22° indicating that it occurred an increase in the crystallinity in PPT2 in relation to the final stage of the process, obtaining of the PPMC. To obtain microcellulose from CSWT, the process behaves as expected, with greater crystallinity in the CSMC. For CPWT biomass, there was a slight reduction in CPMC crystallinity compared to CPT2.

**Table 3:** Crystallinity Indices of Pineapple Peel, Cowpea Pod Skin and Corn Straw^a^

samples	crystallinity index (CI%)
PPWT	40.18
PPT1	58.77
PPT2	67.43
PPMC	73.39
CPWT	36.04
CPT1	52.48
CPT2	70.56
CPMC	69.23
CSWT	45.00
CST1	59.47
CST2	69.87
CSMC	75.00

a Pineapple peel without treatment (PPWT); pineapple peel after acid treatment (PPT1); pineapple peel after basic treatment (PPT2); microcellulose pineapple peel (PPMC); cowpea pod skin without treatment (CPWT); cowpea pod skin after acid treatment (CPT1); cowpea pod skin after basic treatment (CPT2); microcellulose cowpea pod skin; (CPMC); corn straw without treatment (CSWT); corn straw after acid treatment (CST1); corn straw after basic treatment (CST2) and microcellulose corn straw (CSMC).

The PPWT sample presents an index of around 40.18%, and that this value increases considerably after the acid treatment (PPT1) and the basic treatment (PPT2). At the end of the process to obtain MCs (PPMC), a crystallinity index of 73.39% was achieved. The data obtained show higher numbers when compared to those reported in a hydrolysis process without the use of mechanical grinding to extract NC from pineapple peel. Crystallinity indices of 30.72 and 61.19% were reported for fresh samples and nanocellulose, respectively.^30^ However, the tests carried out in this research demonstrate crystallinity lower than that indicated by Pereira et al.,^14^ which reported 24.05% for untreated waste and 80.91% for NC.

For untreated cowpea pod skin (CPWT), there is a CI (%) of around 36.04%. After the acidic (CPT1) and basic (CPT2) treatments, this value undergoes a considerable increase. However, a small decrease in this index was observed after the extraction of MCs, reaching around 69.23%. This behavior is contrary to the expected results and may be an indication that the isolation process was more intense than necessary, reaching crystalline regions of the sample. A decrease in CI% was also observed in the isolation of NC in sisal fibers, where values prior to NFC extraction varied between 67 and 76%, whereas NFC presented crystallinity of 53.6%.^62^

Regarding the crystallinity indexes of the samples for untreated corn straw (CSWT), it appears that it presents an index of around 45.00%, and that this value increases considerably after the acid (CST1) and basic (CST2) treatments. At the end of the process and after extraction of MC (CSMC), the IC value (%) reached 75.00%. Using the chemical-mechanical process for corn cob, a value of 61% for the extracted NC was reported.^21^ A crystallinity index of 25.33% was reported for untreated corn stover and 55.04% for its nanocellulose.^63^ This demonstrates that the treatment used in this research is more efficient than that reported by the aforementioned studies.

After the acid hydrolysis ball milling process, the crystallinity of cellulose can be increased because of the hydrolysis in the bleaching process, which can effectively remove the amorphous phases, lignin and hemicellulose. It was possible to achieve values above 69% for all biomasses, using a mixed extraction methodology, partly chemical and partly mechanical, through the ball mill.

In the process of obtaining NCs using ball milling, NFC was used as a topical hemostatic: *In vitro* the NFC obtained had CI between 64 and 71%.^64^ Furthermore, the use of NC derived from cotton fabric scraps as a cross-linker to reinforce sodium alginate/chitosan hydrogels with a CI of 57.9% was reported.^52^ The CI obtained in this research for cowpea pods skin (CPMC) is within the range of values reported by Mohamed et al.^64^ For PPMC and CSMC, a higher crystallinity index was demonstrated when compared to those reported in the studies, which demonstrates the promising results for possible applications of the nanocelluloses obtained in the study.

In Table 4, a comparison is made between the degree of crystallinity of the microcelluloses and the particle length obtained in this study (PPMC, CPMC, and CSMC) with those reported in the literature, obtained from other biomasses, considering the extraction method, catalyst, and extraction time.

**Table 4:** Comparative Studies on the Production of Nano/Microcellulose via Ball Milling^a^

	input material to ball milling	catalyst	time (h)	crystallinity (%)	size (nm)	refs
1	raw oil palm leaves	chloride-lactic acid deep eutectic solvent (DES)	1.50	47.28	--	(65)
2	avicel	endoglucanase from *Aspergillus niger*	0.90	75	150	(66)
3	commercial cellulose	endo-1–4-β-d-glucanase	1.00	54–59	200–500	(67)
4	paper egg trays	NaOH	4.00	88,7–88,2	300–500	(62)
5	raw sisal fibers	maleic acid	0.50–7.00	53.6	--	(50)
6	hemp hurd	sulfuric acid	4.00–20.00	47–82	196–517	(24)
7	waste cotton	deep eutectic solvents	16.00	57.9	1000–2000	(52)
8	eucalyptus cellulose pulp	*A. niger* A12 strain	0.00–1.50	78.3	294.0	(68)
9	paper sludge	dry/ethanol	6.00	--	340–373	(49)
10	reed *Phragmites australis* straw	NaOH	0.50–6.00	23–51	<10	(69)
11	stalk of coconuts (*Nucifera aurantiaca*)	--	0.16	77.8	55–64	(31)
12	eucalyptus biomass	ethanol	1.00–4.00	17,7–77	131–338	(26)
13	banana peel	DMSO	2.00	77.2	1285	(70)
14	banana bract	DMSO	2.00	75.8	972	(70)
15	cellulose powder	1-butyl-3-methylimidazolium chloride	2.00	65.8	<1000	(71)
16	pineapple peel (PPMC)	sulfuric acid	15.00	73.39	300–600	In this work
17	cowpea pod skin (CPMC)	sulfuric acid	15.00	69.23	30–280	In this work
18	corn straw (CSMC)	sulfuric acid	15.00	75.00	100–250	In this work

a Microcellulose pineapple peel (PPMC); microcellulose cowpea pod skin (CPMC) and microcellulose corn straw (CSMC).

According to Table 4, the crystallinity index of the NC/MC obtained in the present study is higher than previously reported, which may be related to the longer milling time. It is worth highlighting that, apart from the extractions carried out on biomasses such as Avicel,^66^ Reed (*P. Australis*) straw,^69^ and coconut stalks (*N. Aurantiaca*),^31^ the microcelluloses produced in this study exhibit lengths comparable to or even shorter than those reported in other studies. This outcome underscores both the efficiency of the applied method and the potential of the investigated biomass sources.

## Conclusions

Therefore, the results demonstrate the effectiveness of the extraction method applied to the plant residues studied. However, the low yield in the pineapple peel extraction process makes the microcellulose derived from this residue less attractive from an operational point of view. The process for extracting microcellulose from cowpea pod skin residue demonstrated higher yield, in relation to the other biomasses evaluated, which is in agreement with the lower elimination of lignin and hemicellulose fractions. Due to the higher concentration of amorphous fractions, CPMC showed greater thermal stability and a lower degree of crystallinity. The microcellulose extracted from corn straw stood out for presenting a more complete elimination of the lignin and hemicellulose fractions, and consequently, presented a higher degree of crystallinity. All MFCs are in the form of compacted and lamellar materials on a micrometric scale, and the lamellar structures of MCs are constituted by different nanostructures that provide them with unique properties and diverse applications. In addition, the MFCs obtained present promising characteristics to be used as raw material in several applications of the industry. As a suggestion for future work, we recommend the application of MCs as reinforcement material in composites.
